# Molecular hydrogen supplementation in mice ameliorates lipopolysaccharide‐induced loss of interest

**DOI:** 10.1002/pcn5.70000

**Published:** 2024-08-21

**Authors:** Minori Koga, Mayumi Sato, Ryuichi Nakagawa, Shinichi Tokuno, Fumiho Asai, Yuri Maezawa, Masanori Nagamine, Aihide Yoshino, Hiroyuki Toda

**Affiliations:** ^1^ Department of Psychiatry, School of Medicine National Defense Medical College Saitama Japan; ^2^ Graduate School of Health Innovation Kanagawa University of Human Services Kanagawa Japan; ^3^ Department of Bioengineering Graduate School of Engineering Tokyo Japan; ^4^ Division of Behavioral Sciences National Defense Medical College Research Institute Saitama Japan

**Keywords:** blood–brain barrier, inflammation, lipopolysaccharide, molecular hydrogen

## Abstract

**Aim:**

The objective of this study was to evaluate the potential of hydrogen in preventing and treating psychiatric symptoms, particularly depressed mood and loss of interest, and to explore its underlying mechanisms. A mouse model exhibiting inflammation‐derived depressive symptoms was used for the investigation.

**Methods:**

Institute of Cancer Research mice were subjected to a 7‐day intervention of either 30% hydrogen or 40 g per day of air via jelly intake. On the final day, lipopolysaccharide (LPS) was intraperitoneally administered at 5 mg/kg to induce inflammation‐related depressive symptoms. Behavioral and biochemical assessments were conducted 24 h post‐LPS administration.

**Results:**

Following LPS administration, a decrease in spontaneous behavior was observed; however, this effect was mitigated in the group treated with hydrogen. The social interaction test revealed a significant reduction in interactions with unfamiliar mice in the LPS‐treated group, whereas the hydrogen‐treated group exhibited no such decrease. No significant changes were noted in the forced‐swim test for either group. Additionally, the administration of LPS in the hydrogen group did not result in a decrease in zonula occludens‐1, a biochemical marker associated with barrier function at the cerebrovascular barrier and expressed in tight junctions.

**Conclusion:**

Hydrogen administration demonstrated a preventive effect against the LPS‐induced loss of interest, suggesting a potential role in symptom prevention. However, it did not exhibit a suppressive effect on depressive symptoms in this particular model. These findings highlight the nuanced impact of hydrogen in the context of inflammation‐induced psychiatric symptoms, indicating potential avenues for further exploration and research.

## Graphical abstract

This study explores hydrogen's potential to prevent lipopolysaccharide (LPS)‐induced loss of interest in a mouse model of inflammation‐derived depressive symptoms. Hydrogen administration mitigated the decrease in spontaneous behavior post‐LPS and maintained normal social interaction, suggesting a preventive role against LPS‐induced interest loss.

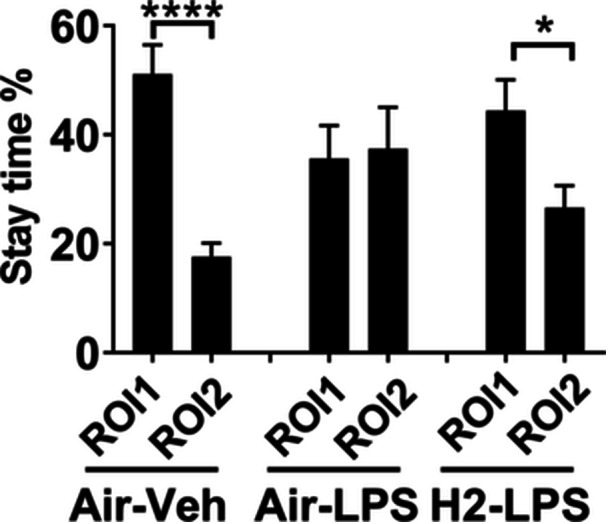

## INTRODUCTION

Oxidation is inevitable for organisms that gain energy through aerobic respiration. However, chronic oxidative accumulation, known as oxidative stress, impairs cell properties and morphology maintenance and causes health problems, such as heart disease and angiopathy.^1^, ^2^, ^3^ In humans, the brain accounts for only 2% of body weight but consumes up to 20% of the total oxygen and a large amount of energy. Therefore, the brain is at high risk for oxidative stress. Various factors contribute to oxidative stress, including superoxide anion radicals, hydrogen peroxide, and hydroxyl radicals. The most prevalent in vivo factor is superoxide, which is counteracted by superoxide dismutase.^4^ Hydrogen peroxide is detoxified by catalase and glutathione peroxide. Carotenoids, such as α‐ and β‐carotene, which are ingested, effectively remove singlet oxygen.^5^ Hydrogen is a simple reducing molecule that has attracted significant research interest, as accumulating evidence supports the contribution of hydrogen to physical health. Hydrogen intake has been recently shown to exert preventive or therapeutic effects against physical diseases and central nervous system‐related diseases.^6^, ^7^ However, the mechanism by which hydrogen prevents the onset and progression of these diseases has not yet been clarified.

Oxidative stress induces inflammation,^8^, ^9^ and vice versa.^10^ Systemic administration of lipopolysaccharide (LPS),^11^ which causes behavioral abnormalities similar to the core symptoms of depression (such as anxiety, depression, and lack of interest), induces cytokine release and increases reactive oxygen species production. These factors may contribute to the pathophysiology of psychiatric symptoms.

The aim of this study was to analyze the effect of hydrogen administration on restoring behavioral abnormalities in LPS‐induced oxidative stress mice models. Furthermore, to elucidate the biochemical mechanism related to this effect, differences in protein expression in the brain and peripheral blood were assessed. Given that microglia and astrocytes have been implicated in the brain immune system,^12^, ^13^ the following markers were investigated: glial fibrillary acidic protein (GFAP), an astrocyte marker; ionized calcium‐binding adapter molecule 1 (IBA1), a marker for activated microglia; and peripheral benzodiazepine receptor (PBR), a marker of glial activation that contributes to inflammation. Additionally, because LPS‐induced systemic inflammation has been reported to disrupt the blood–brain barrier (BBB),^14^ both zonula occludens‐1 (ZO‐1) and occludin were investigated. ZO‐1 is located on the cytoplasmic membrane surface of intercellular tight junctions and may be involved in signal transduction at cell–cell junctions.^15^, ^16^ Occludin is the first component of the tight junction to be identified and may contribute to barrier and fence functions or regulation.^17^, ^18^, ^19^ The findings of this study could contribute to the prevention of the onset of mental disorders, the development of treatments, and drug discovery for these disorders.

## MATERIALS AND METHODS

### Animals

Eight‐week‐old male Institute of Cancer Research (ICR; CD‐1) mice were purchased from Japan SLC (Hamamatsu, Japan) and maintained in a temperature (22–24°C)‐ and humidity (50%)‐conditioned room with a 12:12 h light–dark cycle, with lights on at 7:00 AM. Only male mice were used in behavioral and biochemical analyses to eliminate the influence of the estrous cycle. Food and water were provided ad libitum. The mice were housed (maximum of four mice per cage) in standard cages, measuring 225 × 338 × 140 mm, with sawdust. Additionally, two mice were prepared and used as social targets for the social interaction test. Sixteen mice per experiment were habituated in home cages in the animal facility for 1 week after arrival. After habituation, water supply bottles were replaced with jelly containing 30–40 mg/L air bubbles (Shinryo, Tokyo, Japan) for 1 day.

### Jelly supplementation

Jellies containing air or molecular hydrogen at 30–40 mg/L were used; the composition is shown in Table 1. To determine the amount of jelly consumed by mice, nine mice were prepared in individual cages. They were dehydrated for 24 h by removing the water bottles, after which 40 g of jelly containing air bubbles was placed in a 10‐cm dish; the jelly was weighed after 0, 30, 60, 120, 180, 300, and 420 min. Another dish was placed in the same room with the jelly and the mouse cage, and the weight of the jelly at each time point was measured to determine weight loss due to drying. The amount of jelly consumed by the mice was defined as the weight loss of the jelly in the mouse cage at each time point minus the weight loss due to drying.

**Table 1:** Composition of jelly administered to mice.

Hydrogen gel		Placebo gel	
Ingredients	Composition	Ingredients	Composition
Water	98.1%	Water	98.1%
Gelator*	1.7%	Gelator*	1.7%
Sucrose myristate	0.1%	Sucrose myristate	0.1%
Acesulfame potassium	0.1%	Acesulfame potassium	0.1%
Molecular hydrogen	~30–40 mg/L	Air	~30–40 mg/L
*Gelator			
**Ingredients**	**Composition**		
Carrageenan	20%		
Xanthan gum	15%		
Tara gum	10%		
Carob bean gum	5%		
Glucomannan	5%		
Potassium dihydrogen phosphate	10%		
Dextrin	35%		

Hydrogen was administered to the mice for 7 days prior to the induction of neuroinflammation with LPS. This duration was selected based on previous studies that demonstrated the significant neuroprotective effects of hydrogen when administered for similar periods.^20^, ^21^, ^22^, ^23^ Hydrogen is commonly consumed as a health supplement, suggesting potential preventive benefits. Therefore, hydrogen was administered to evaluate its preventive effects before inducing neuroinflammation.

### Group assignment and treatments

The mice were assigned to three groups: Group 1 mice (control group) were administered jelly containing air bubbles for 1 week; then, saline was administered intraperitoneally on the last day of the week. Group 2 mice (LPS group) were administered jelly containing air bubbles for 1 week, and on the last day of the week, LPS derived from *Escherichia coli* serotype O127: B8 (Sigma, St. Louis, MO, USA) was administered at a dose of 5 mg per kg body weight to induce systemic inflammation, which was used as a pathological model of psychiatric symptoms based on the neuroinflammation hypothesis. Group 3 mice (H_2_‐LPS group) were administered jelly containing hydrogen bubbles (Shinryo) for 1 week, and on the last day of the week, LPS was administered at a dose of 5 mg/kg. Jelly (40 g) with air or hydrogen bubbles was supplied daily on the stainless‐steel mouse cage lid top. Saline and LPS were injected at a fixed volume of 10 mL/kg body weight. A social interaction test was performed 24 h after LPS injection, and open‐field and forced‐swim tests were conducted the next day.

LPS (5 mg/kg) was administered intraperitoneally to induce neuroinflammation. This dose was selected based on previous studies modeling neuroinflammation, which reported behavioral experiments conducted 24 h after LPS administration.^24^ According to Dantzer et al.,^25^ inflammation transitions to sickness and depression 24 h after LPS administration, following an initial 2–6‐h period of acute inflammation. Therefore, behavioral tests and tissue collection for gene expression analysis were performed 24 h post LPS administration to observe behavioral abnormalities caused by inflammation rather than acute motor function decline.

### Tissue collection

**Figure 1:**
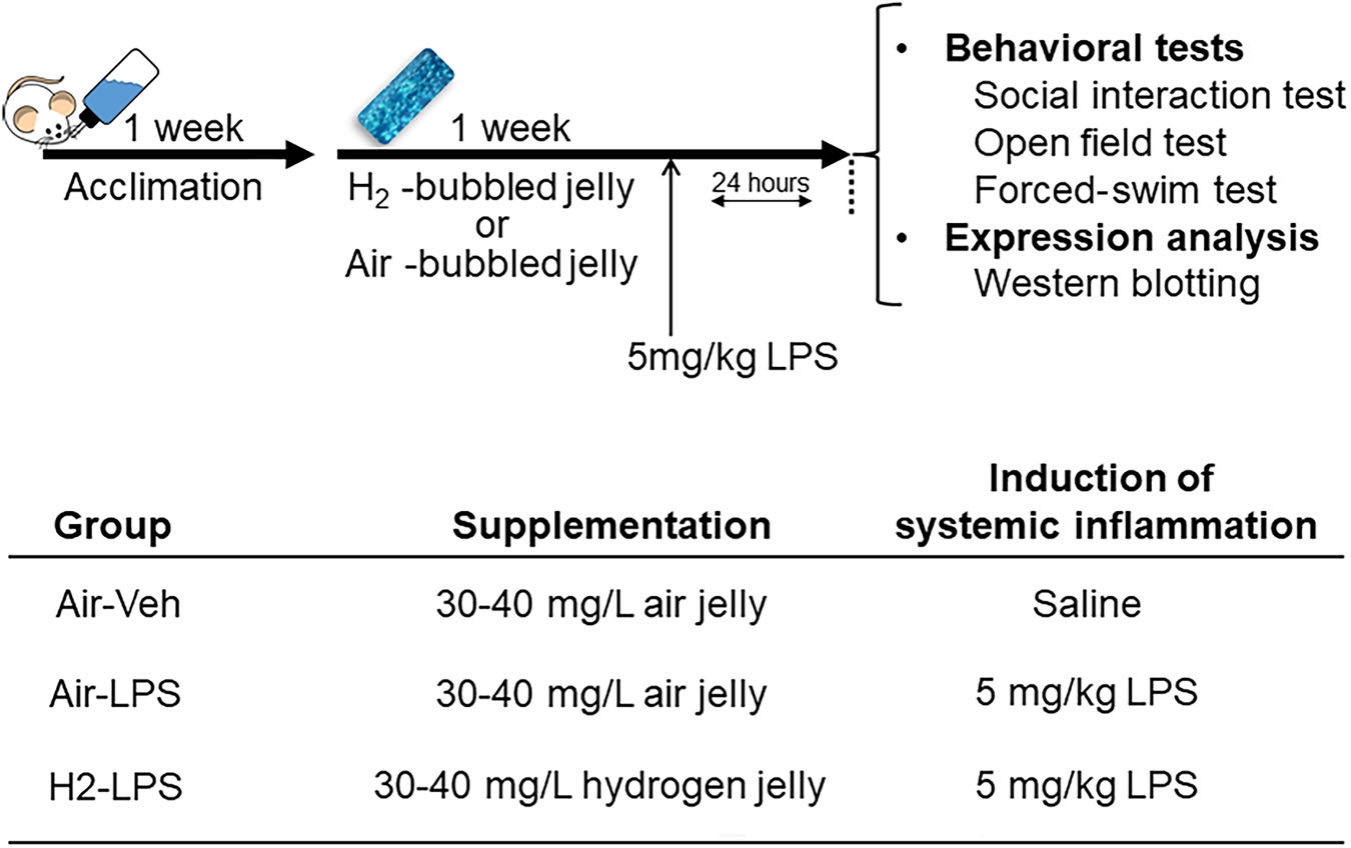
Schematic of the experimental design.

After the behavioral tests, the mice were anesthetized using an anesthetic mixture (0.75 mg/kg medetomidine [Medetomin injection, Meiji Seika Pharma], 4.0 mg/kg midazolam [midazolam injection, TEVA, Takeda Pharmaceutical], and 5 mg/kg butorphanol [Vetorphale, Meiji Seika Pharma]) and cardio‐perfusion with 0.1 M phosphate‐buffered saline (PBS) to remove blood. The anesthetic mixture was injected at a volume of 10 mL/kg body weight. Next, the brains were extracted and immersed in an RNAlater solution (Thermo Fisher Scientific). The brains were dissected the following day to obtain the hippocampus for protein expression analysis. The dissected tissues were stored at −80°C until analysis. The schedule of the treatment, behavioral tests, and tissue sampling for protein expression analysis are shown in Figure 1.

In Groups 1 and 3, individuals with severe injuries from fights were removed (one individual from each group). An injury was discovered in one mouse in Group 2 after the sociability test, and so that mouse was subsequently removed from the study.

All procedures were performed in accordance with ARRIVE guidelines and the National Institutes of Health Guide for the Care and Use of Laboratory Animals (NIH Publications No. 8023, revised 1978). All animal experiments were approved by the local Animal Investigation Committee of the National Defense Medical College (Approval numbers: 18008 and 22042).

### Behavioral tests

#### Sociability test

Twenty‐four hours after the last injection, sociability was measured using a U‐shaped two‐choice field.^26^ This was used to assess the behavioral states of social approaching and interest by determining the amount of time the experimental mice spent and interacted with a social stimulus mouse in the “social area” versus the “non‐social area” of the field. The U‐shaped two‐choice field was a modified open field (40 cm in width, 30 cm in depth, and 30 cm in height) that was partially partitioned with a wall (1 cm in thickness, 18.5 cm in width, and 28.5 cm in height) to the central point, such that the field contained two closed quadrants and two open quadrants of the same size. The upper left and right corners of each area contained two small metal‐meshed chambers (10 cm wide, 6 cm deep, and 6 cm high), and the chamber in the upper left corner was used to house the social stimulus mouse. A 10–12‐week‐old male ICR mouse was used as the social stimulus. The test mice were allowed to move freely without the socially stimulated mice in any of the chambers for 5 min to acclimatize to the field. The mice were then allowed to move freely in the upper left chamber for 5 min with the socially stimulated mice. The interaction area was defined as the area 3.3 cm in front and 5 cm to the side of each chamber (the left area was defined as region of interest [ROI] 1, and the right area was defined as ROI 2), and the total time spent in these areas was measured for each test mouse. The ratio of time spent in ROI 1 and ROI 2 to the session time (300 s) for each mouse was then calculated.

#### Open‐field test

The following morning, an open‐field test was conducted to assess spontaneous locomotor activity levels, anxiety, and willingness to move in an unfamiliar environment. The assessment was performed in an open‐field arena comprising an acrylic box measuring 43.2 × 43.2 × 30.5 cm (Med Associates). The sidewalls were covered with cardboard to obstruct the open field to mice. Horizontal and vertical arrays of 16 infrared beams tracked the horizontal and vertical movements, respectively. The mice were placed in the center of the field and allowed to freely explore the chamber for 5 min in a brightly lit room. Feces and urine were removed from the arena, and the arena was cleaned using 20% ethanol after each recording. The Activity Monitor Version 7.0.5.10 software (Med Associates) was used to analyze the total movement distance and rearing activity of the mice.

#### Forced‐swim test

In the afternoon, after the open‐field test, the forced‐swim test was conducted according to the methodology outlined by Porsolt et al.^27^ However, MicroAct (NeuroScience) was used to measure the duration of immobility. Promptly before the test, a small magnet (1.6 mm in diameter and 0.5 mm in thickness) was attached to the heels of both feet using instant adhesives. Each mouse with attached magnets was placed, for 6 min, in an acrylic cylinder (18 cm in height and 11 cm in diameter) filled with water (maintained at 22–23°C) to a depth of 12.5 cm, which was surrounded by round coils. Small electric currents were generated in the coils according to the movement of the magnet attached to the heels. This current was amplified, changed into a voltage, and recorded by the system. The duration of immobility was measured during the last 5 min of the 6‐min testing period. The duration of immobility was automatically detected. The system was adjusted to detect the voltage pulse within the frequency range of 1–30 Hz and with an amplitude over 0.10 V. Changes in voltage reflecting swimming behavior were analyzed using these settings. The duration of immobility was defined as the total time of each interval (s) over 1.00 s between the detected adjacent pulses.

### Western blotting analysis

The extracted hippocampus was homogenized using the Polytron handheld homogenizer (PT1200E, Kinematica AG) in PBS with 0.5% sodium sarcosine and 1 × Halt Protease Inhibitor Cocktail (Thermo Fisher Scientific). The homogenate was centrifuged at 15,000 r.p.m. for 10 min, and the supernatant was separated and used for Western blotting. The concentration of the protein samples was determined using a bicinchoninic acid kit (Thermo Fisher Scientific) and diluted with PBS to adjust the concentration to 4 µg/µL. Invitrogen NuPAGE LDS sample buffer (4×; Thermo Fisher Scientific) with 4 mM dithiothreitol (FUJIFILM Wako Pure Chemical Corporation, Osaka, Japan) was mixed with the diluted samples to obtain a 1× concentration. The final concentration of each sample was 3 µg/µL. Protein samples were then heated at 70°C for 10 min. Next, 30 µg of protein samples were separated on 4%–10% gradient sodium dodecyl sulfate‐polyacrylamide gels (Bio‐Rad) and transferred to Immobilon‐P polyvinylidene difluoride membranes (Merck Millipore). After electrophoresis, membranes were washed in PBS with 0.5% Tween 20, then transferred into 5% Blocking One blocking solution (Nacalai Tesque) for 1 h. Membranes were probed with primary antibodies against GFAP (ab53554, 1:10,000, Abcam), IBA1 (ab5076, 1:200, Abcam), PBR (ab109497, 1:200, Abcam), occludin (13409‐1‐AP, 1:2,000, ProteinTech), ZO‐1 (Ab221547, 1:2,000, Abcam), and α‐tubulin (1:50,000, FUJIFILM Wako Pure Chemical Corporation) at 4°C overnight. The next day, membranes were washed to remove excess antibodies and probed with horseradish peroxidase (HRP)‐conjugated secondary antibodies against rabbit IgG (Cytiva) for occludin and ZO‐1 and mouse IgG for β‐actin for 1 h. Next, the membranes were washed with PBS containing 0.5% Tween 20 (FUJIFILM Wako Pure Chemical Corporation) to remove excess antibodies. Luminescence was detected using the Amersham Imager 600 (Cytiva) using ImmunoStar LD chemiluminescent HRP substrate (FUJIFILM Wako Pure Chemical Corporation) and quantified using the Image Studio Lite Version 5.2 software (LICOR Biosciences). All detected quantities were normalized to α‐tubulin and used for group comparison analysis.

### Enzyme‐linked immunosorbent assay

Mouse plasma interleukin (IL)−1β and tumor necrosis factor (TNF)‐α concentrations were determined using a commercially available enzyme‐linked immunosorbent assay (ELISA) kit (Mouse IL‐1 beta/IL‐1F2 Quantikine ELISA Kit, Mouse TNF‐alpha Quantikine ELISA Kit, R&D Systems). The serum was then diluted in PBS at a ratio of 1:3.3. Subsequently, 50 µL of the diluted plasma was dispensed onto coated ELISA plates, and IL‐1β and TNF‐α levels were examined in the samples according to the manufacturer's instructions. Each sample was assayed in duplicates.

### Statistical analyses

Changes in body weight before and after treatment with air or hydrogen jelly intake were measured using repeated‐measures analysis of variance (rmANOVA). The unpaired *t*‐test was used to compare two experimental groups and Dunnett's test for three groups. The quantile range outliers method and rmANOVA were conducted using JMP 15.2.0 (SAS Institute), and unpaired *t*‐test and Dunnett's test were performed using GraphPad Prism 9 (GraphPad Software). The data are expressed as mean ± standard error of the mean and were incorporated into figures using GraphPad Prism 9. A *p*‐value < 0.1 was considered to trend toward statistical significance, and *p* < 0.05 was considered statistically significant.

## RESULTS

### Quantity of jelly consumption

**Figure 2:**
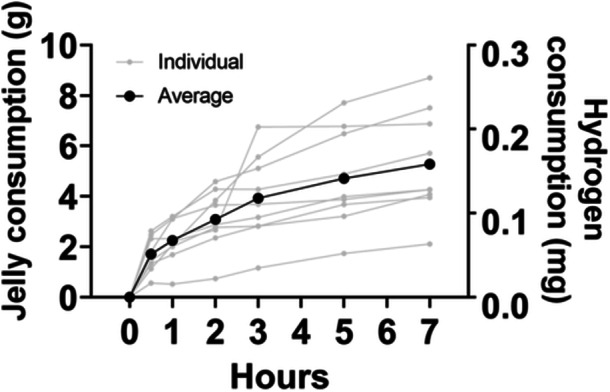
Jelly consumption by mice. Gray lines indicate the individual amount of jelly consumption, and the black line indicates the average consumption at 0, 30, 60, 120, 180, 300, and 420 min after the jelly had been placed in the mouse cages. No outlier values existed in the amount of jelly consumption at any time point.

Jelly consumption of mice over a 7‐h period was assessed. Figure 2 indicates the amount of jelly consumed by the mice at each time point. No outliers were found for consumption at any time point for the nine mice. After 7 h of administration, the average jelly consumption was 5.28 ± 2.08 g (0.16 ± 0.06 mg as the amount of gas). The lowest and highest consumption was 2.11 g and 8.70 g, respectively (0.063 mg and 0.261 mg, respectively, as the amount of gas).

### Influence of hydrogen intake on body weight

**Figure 3:**
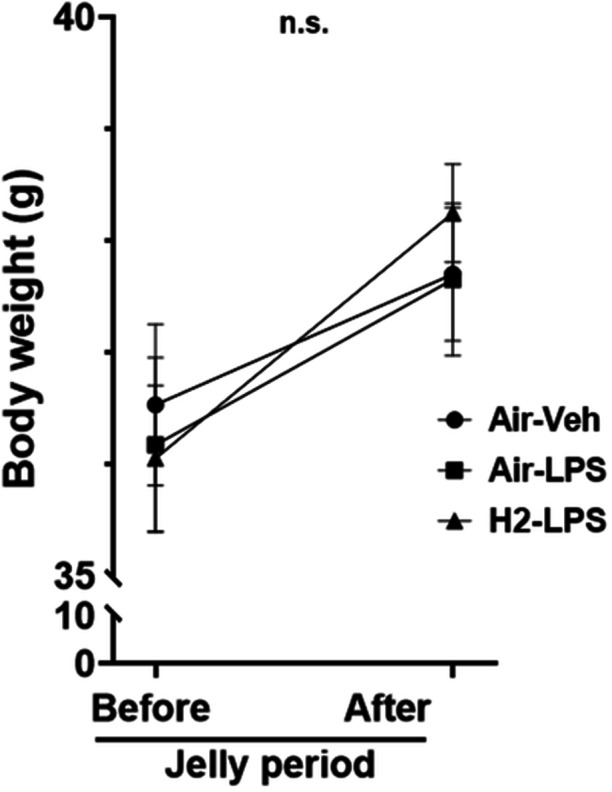
Changes in body weight of mice before and after 1 week of air or hydrogen jelly supplementation. No significant differences (n.s.) were observed among the groups using repeated‐measures analysis of variance (*n* = 15 for each group, *p* = 0.51).

After 7 days of hydrogen jelly intake, no significant difference in body weight was observed between the hydrogen and air jelly mice groups (rmANOVA; Figure 3).

### Social interaction

**Figure 4:**
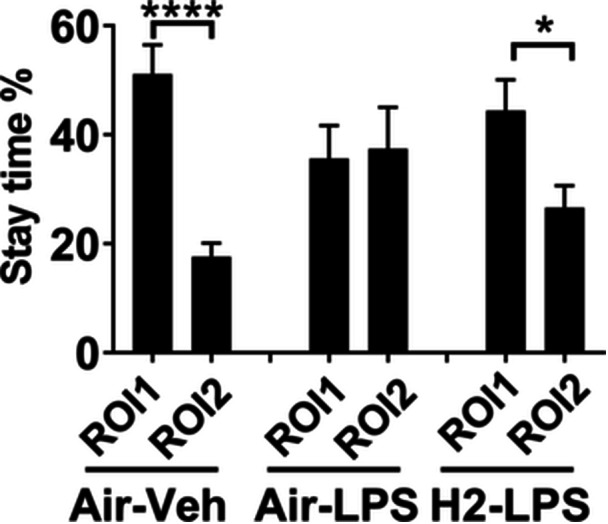
Effect of hydrogen intake on social interaction. Mice received air‐ or hydrogen‐bubbled jelly for 1 week, followed by 5 mg/kg lipopolysaccharide (LPS) to induce systemic inflammation. Interest was assessed using the social interaction test. The means ± standard errors of the percentage of time spent in region of interest (ROI) 1 and ROI 2 relative to the total session time (300 s) for each mouse in the three experimental groups are indicated. n.s., not significant; ***p < 0.001 and ****p < 0.0001 vs. the Air–Veh group using the *t*‐test.

Figure 4 shows the percentage of time spent in the ROI 1 and ROI 2 areas during a 5‐min session in the social interaction test. Mice spent significantly more time in the social areas than in the unsocial areas because they interacted with each other when mice of the same species were present. Thus, the control group spent a greater percentage of time in ROI 1 relative to the test time (51.06% ± 5.42% in ROI 1 and 17.62% ± 2.47% in ROI 2, *t*_(28)_ = 5.612, *p* < 0.0001, *t*‐test). LPS administration reduced the uneven distribution in ROI 1 (35.59% ± 6.08% in ROI 1 and 37.44% ± 7.60% in ROI 2, *t*_(30)_ = 0.1894, not significant, *t*‐test). In contrast, hydrogen intake recovered the LPS‐induced reduction and mirrored the results observed in the control group (44.43% ± 5.65% in ROI 1 and 26.58% ± 4.07% in ROI 2, *t*_(28)_ = 2.564, *p* < 0.05, *t*‐test).

### Spontaneous activity

**Figure 5:**
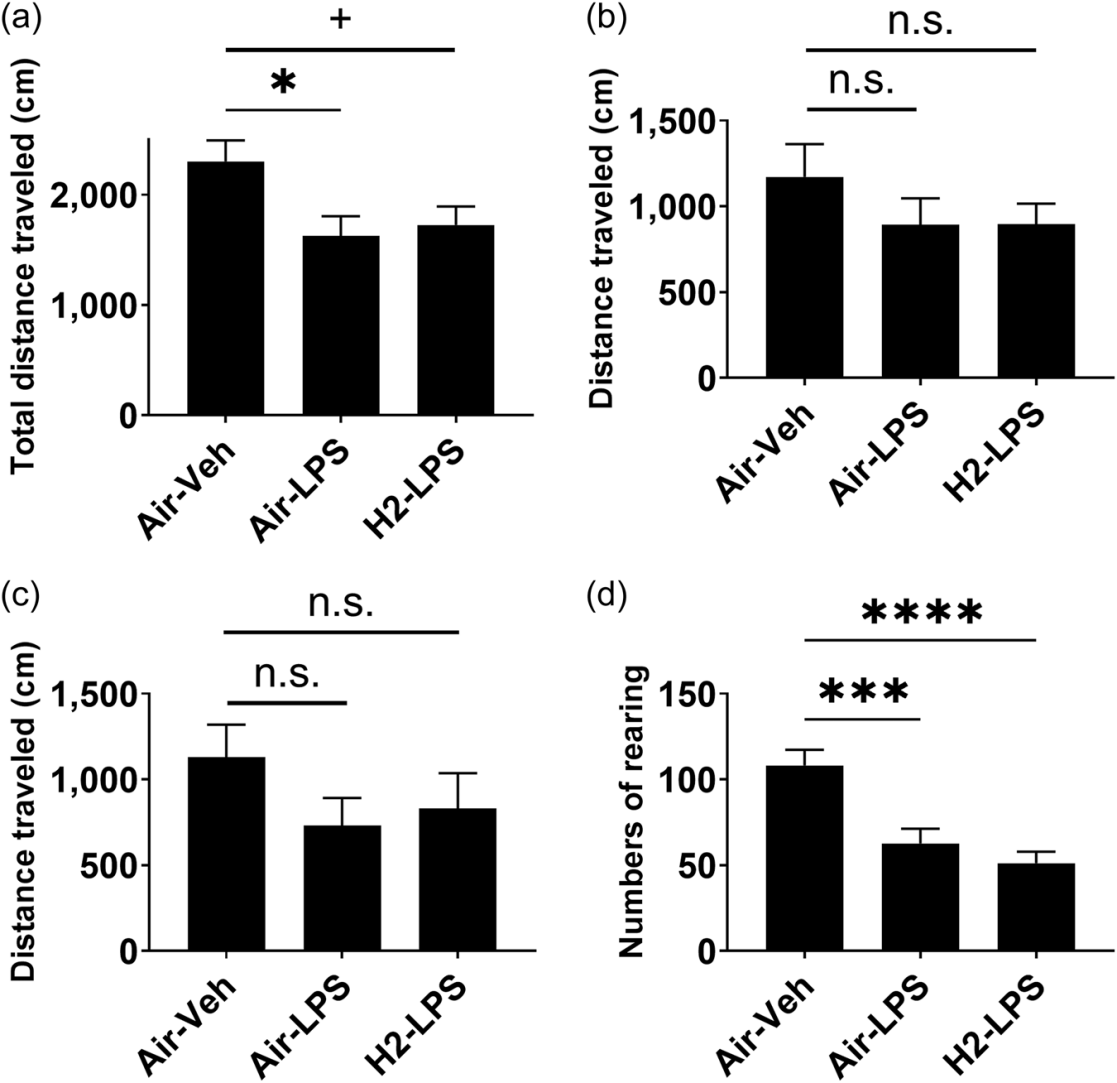
Effect of hydrogen intake on spontaneous activities. Mice received air or hydrogen‐bubbled jelly for 1 week, followed by 5 mg/kg lipopolysaccharide (LPS) to induce systemic inflammation. Spontaneous activity was assessed using an open‐field test. The average ± standard error of the (a) total distance, (b) distance traveled in the center area, (c) distance traveled in the outer zone, and (d) number of rearing are indicated. n.s. not significant, +*p* < 0.1, *p < 0.05, ***p < 0.001, and ****p < 0.0001 vs. Air–Veh group using Dunnett's test.

The total distance decreased significantly in the Air‐LPS group (1625 ± 180.9 cm, *p* < 0.05) compared to the Air–Veh group (2303 ± 190.0 cm). However, the H_2_‐LPS group showed a decreasing trend in the total distance (1727 ± 167.7 cm, *p* < 0.1); thus, hydrogen supplementation could not significantly reverse the decrease in activity compared with that in the Air–Veh group (Figure 5a). The distance in the center and outer zones indicated no significant differences among the groups (Figure 5b,c). The vertical activity was significantly reduced in the Air‐LPS group (62.73 ± 8.638 times, *p* < 0.001) compared to the Air–Veh group (108.2 ± 9.108 times). However, the H_2_‐LPS group showed a significant reduction (51.13 ± 6.777 times, *p* < 0.0001); thus, hydrogen supplementation did not reverse the LPS‐induced reduction (Figure 5d).

### Depressiveness

**Figure 6:**
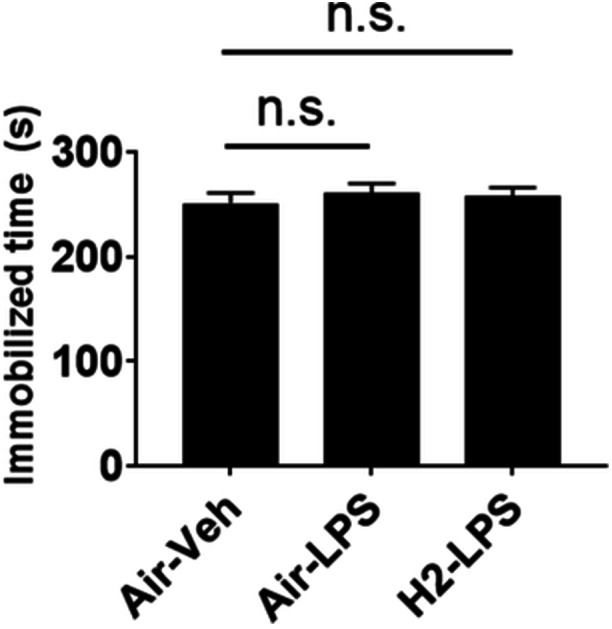
Effect of hydrogen intake on immobility in the forced‐swim test. Mice received air or hydrogen‐bubbled jelly for 1 week and then 5 mg/kg lipopolysaccharide (LPS) to induce systemic inflammation. Their depressed mood was assessed using the forced‐swim test. The duration of immobility in the last 5 min of the 6‐min session is indicated as the average ± standard error. n.s., not significant vs. the Air–Veh group using Dunnett's test.

No significant difference was observed in immobility time during the last 5 min of the 6‐min session in the forced‐swim test among all groups (Figure 6).

### Hydrogen‐intake‐induced expression changes in glial and tight junction marker proteins in brain tissue

**Figure 7:**
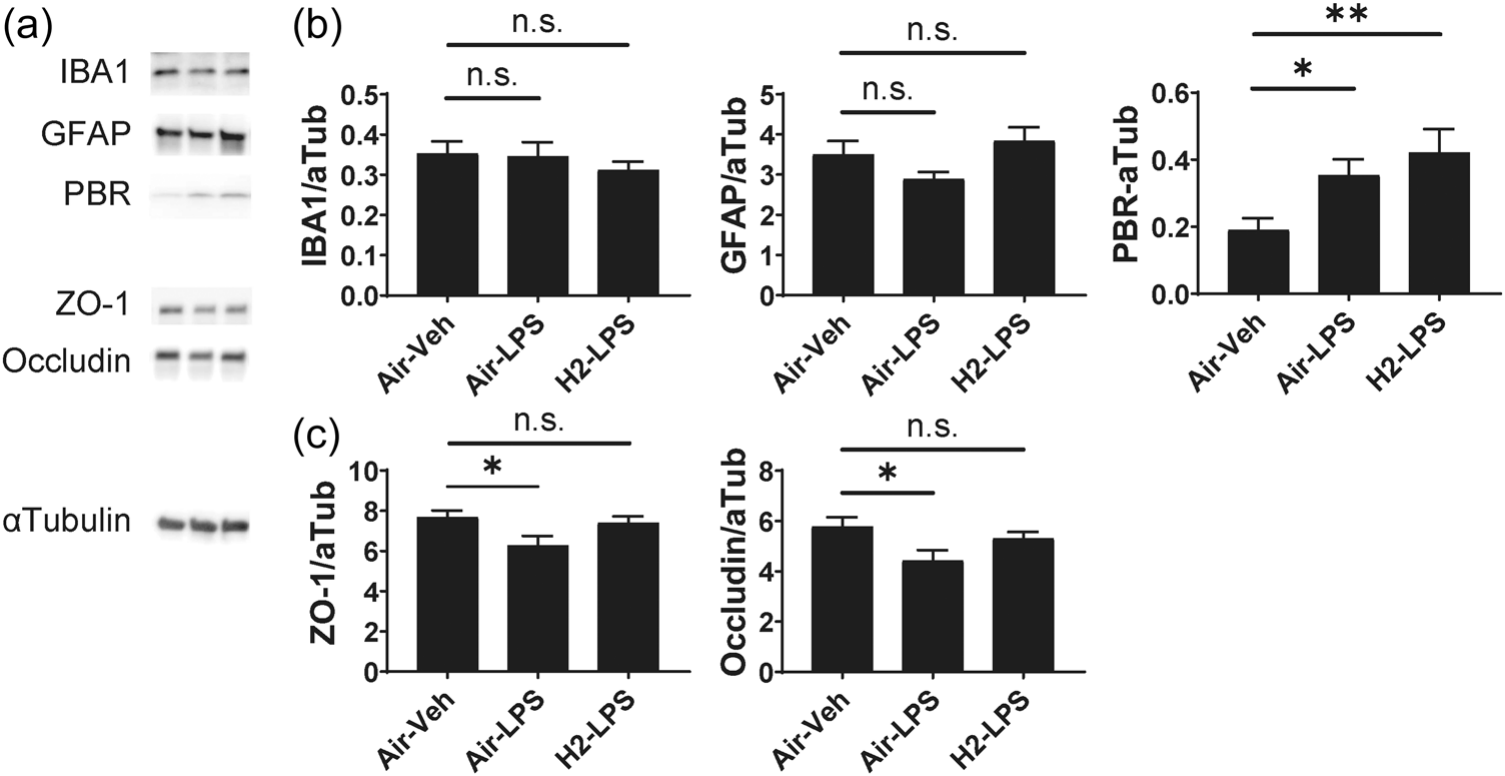
Protein expression in the hippocampus after lipopolysaccharide (LPS) administration. (a) Representative images of western blots are shown on the left side. The bar graphs indicate the average ± standard error ratio of the intensity of detected band images of (b) glia‐related molecules, ionized calcium‐binding adapter molecule 1, glial fibrillary acidic protein, and peripheral benzodiazepine receptor, and (c) barrier function markers of the blood–brain barrier, zonula occludens‐1 and occludin, normalized to that of α‐tubulin in the mice from the three experimental groups. n.s., not significant; *p < 0.05 and **p < 0.01 vs. Air–Veh group using Dunnett's test.

Figure 7a shows representative western blotting images. Figure 7b indicates the average intensity of the target proteins GFAP, IBA1, and PBR, which are markers for astrocyte, microglia, and activated glial cells, respectively; the intensity was normalized to α‐tubulin. GFAP and IBA1 expression did not differ significantly among any of the groups. Hydrogen intake did not reduce the expression of PBR, which contributes to LPS‐increased inflammation. Figure 7c indicates the average intensity of the markers of BBB function normalized to α‐tubulin. ZO‐1 and occludin showed significantly lower expression levels in the LPS group than in the control group. However, hydrogen intake restored their expression to normal levels.

### Hydrogen‐intake‐induced expression changes in inflammatory cytokines in the serum

**Figure 8:**
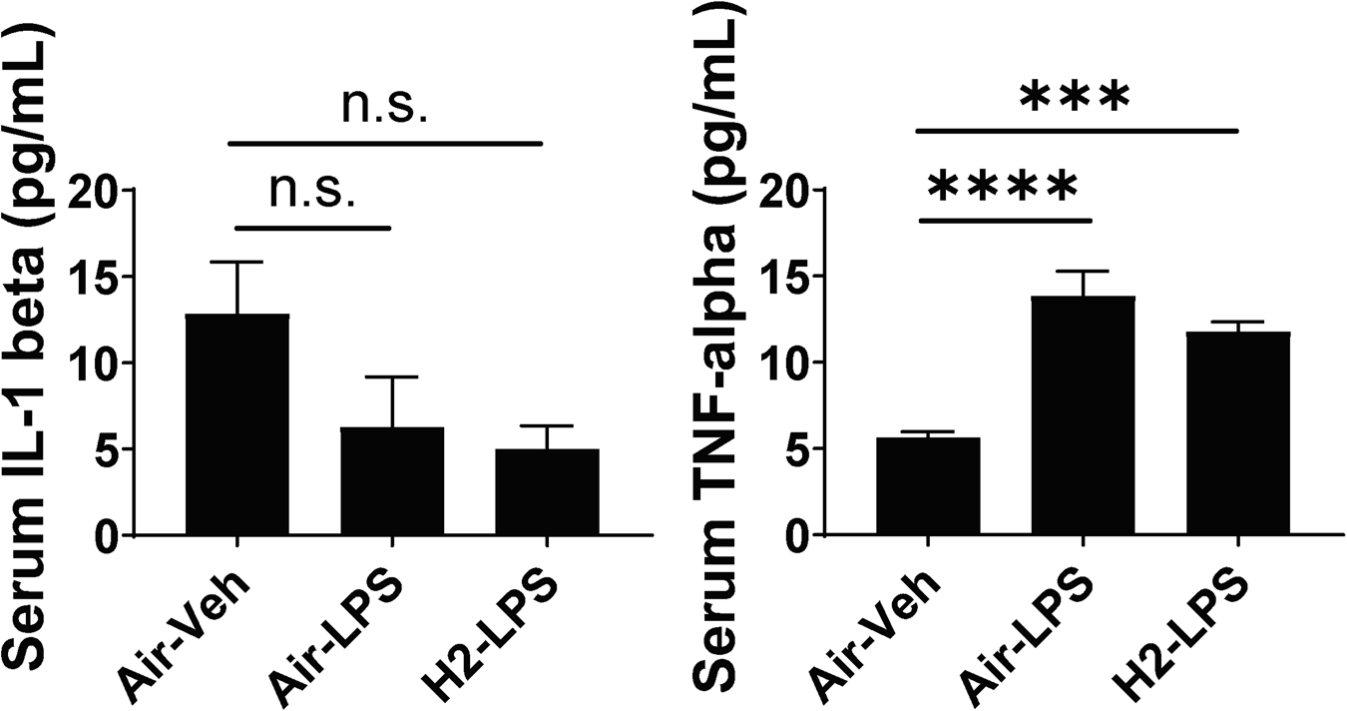
Protein concentration in the serum after lipopolysaccharide (LPS) administration. The bar graphs indicate the serum interleukin (IL)‐1β and tumor necrosis factor (TNF)‐α concentrations of the mice analyzed using enzyme‐linked immunosorbent assays. n.s.: not significant, ***p < 0.001 and ****p < 0.0001 vs. the Air–Veh group using Dunnett's test.

Figure 8 shows the concentration of inflammatory cytokines in the mice serum derived after behavioral tests in the three groups. IL‐1β levels did not differ significantly among the three groups. TNF‐α levels were significantly increased by LPS‐induced systemic inflammation and could not be reversed by hydrogen intake.

## DISCUSSION

This study investigated the preventive effect of hydrogen intake on the development of psychiatric disorders and explored the underlying mechanisms. The reduction in interest (reduced interaction with the same mouse species) caused by LPS‐induced systemic inflammation was inhibited by hydrogen administration. In addition, the biochemical analysis in the present study suggested that hydrogen might have a mitigating effect on the LPS‐induced decrease in protein expression of BBB markers.

In the open‐field test in the present study, LPS induced a reduction in spontaneous activity and exploratory behavior, which can be interpreted as a decrease in motivation. However, hydrogen administration did not suppress these effects. Although hydrogen may not affect the decrease in motivation, we used a high dose (5 mg/kg) to create a neuroinflammation model. Consequently, a strong inflammatory response occurred, and the hydrogen dosage used in this study might have been insufficient for recovery. In contrast, the social interaction test showed that LPS‐induced reduction in interaction was clearly suppressed by hydrogen administration. This result suggests that the decrease in motivation and interest may involve different mechanisms.

Biochemical analysis revealed significant decreases in ZO‐1 and occludin, components of the BBB between the control and LPS groups. In contrast, no significant difference was observed between the control and hydrogen + LPS groups, suggesting a protective effect of hydrogen against LPS‐induced BBB damage. Previous studies have implicated BBB abnormalities in the pathogenesis of psychiatric disorders.^28^, ^29^, ^30^, ^31^ The findings of the present study suggest that hydrogen has a restorative effect on the BBB, reversing LPS‐induced weakening of its gatekeeper functions. However, as there was no significant difference between the LPS and hydrogen + LPS groups in this study, we concluded that the evidence for hydrogen's protective effect on BBB damage is weak. Given the weak evidence for BBB protection, it is crucial to explore other potential mechanisms by which hydrogen might exert its effects. We did not observe anti‐inflammatory effects of hydrogen administration, suggesting that inflammation is not a factor in behavioral abnormalities and their suppression. Given that a neuroinflammation hypothesis of psychiatric disorders has been proposed, intervention studies with anti‐inflammatory agents in patients with psychiatric disorders have been conducted, but the results have been mixed.^32^, ^33^ Overall, anti‐inflammatory agents have not been conclusively shown to be effective in treating psychiatric disorders, suggesting that inflammation may be an epiphenomenon of psychiatric disorders. One possible mechanism for the behavioral improvement with hydrogen administration is its role as an antioxidant. Inflammation and oxidative stress are closely related, with each capable of elevating the other. Our results of western blotting showed no increase in the expression of IBA1 in the hippocampus of mice subjected to LPS‐induced inflammation. As noted by Shi et al.,^34^ the expression of IBA1 in brain immune cells in response to LPS is inconsistent. It may be that PBR is a more appropriate marker for reflecting neuroinflammation. Given that LPS‐induced inflammation was not suppressed in this study, it is possible that hydrogen suppressed oxidative stress caused by inflammation. Oxidative lipids and oxidative stress disrupt the BBB.^35^ Therefore, LPS‐induced inflammation may have triggered oxidative stress, leading to BBB disruption.

This study had several limitations. First, the jellies were provided ad libitum; therefore, the dosage for each mouse varied. Nevertheless, oral administration of hydrogen ad libitum in hydrogen‐rich water has been commonly used in previous reports, and the biochemical and behavioral effects of molecular hydrogen have been evaluated. Previous studies using hydrogen‐rich water have generally administered hydrogen‐rich water at 0.8–3 mg/L concentrations.^36^, ^37^, ^38^, ^39^, ^40^, ^41^, ^42^, ^43^, ^44^, ^45^ Recently, Li et al.^42^ demonstrated the protective effect of hydrogen‐rich water against liver damage in a fatty liver model by administering a high concentration of 7.0 mg/L hydrogen. Although the daily water intake of mice depends on the mouse species and week age, it is generally ˂8 mL.^46^ In the study reported by Li et al., this resulted in a daily hydrogen intake of approximately 0.056 mg. In contrast, the mice in the present study received, at a minimum, 0.063 mg of hydrogen per day through the bubble‐containing jelly. Therefore, despite the variation inherent in ad‐libitum intake, it seems likely that the hydrogen dose exceeded that of most previous studies.

Second, the causal relationship between behavioral changes and BBB status changes after hydrogen administration in the systemic inflammation models has not been fully clarified; therefore, further investigation is required. Third, this study only tested a single dose of LPS in the neuroinflammation model. Consequently, we were unable to determine whether the observed effects were due to simple sickness behavior or inflammation‐induced neurodegeneration. Fourth, only an LPS model was used in this study. Abnormal accumulation of oxidative stress and/or inflammation has been reported in pathological models of psychiatric disorders, such as chronic mild stress and social defeat stress.^47^, ^48^, ^49^, ^50^ It is necessary to examine these stress models to determine whether hydrogen intake effectively suppresses the onset of psychiatric symptoms and investigate the pathological pathway classifications. Fifth, novel explorations into the mechanisms of LPS‐induced BBB decay and BBB protection by hydrogen ingestion are necessary because the findings of this study and previous studies suggest a cytokine‐release‐independent pathway in macrophages and microglia. Sixth, we used total tissue lysates for the western blotting analysis because of the high cell specificity of the target proteins. While this method allows for the detection of proteins from multiple cell types, it may not provide a detailed understanding of the precise cellular mechanisms involved. For a more accurate elucidation of these mechanisms, future studies should consider isolating and analyzing proteins from individual cell types.

As there are still no definitive therapeutic or preventive agents for psychiatric disorders, elucidation of the biological mechanisms underlying psychiatric disorders is crucial. The findings of this study and previous studies suggest that hydrogen has a specific effect in protecting the BBB rather than having a general antioxidant and anti‐inflammatory effect. Further investigation could help elucidate disease pathogenesis and facilitate the treatment or prevention of psychiatric symptoms.

## AUTHOR CONTRIBUTIONS

Mayumi Sato, Minori Koga, Ryuichi Nakagawa, Fumiho Asai, and Yuri Maezawa performed the experiments. Mayumi Sato, Minori Koga, and Fumiho Asai performed the data analysis. Mayumi Sato and Minori Koga wrote the original draft of the manuscript. Minori Koga, Shinichi Tokuno, and Masanori Nagamine reviewed and edited the manuscript. Hiroyuki Toda and Aihide Yoshino supervised the study.

## CONFLICT OF INTEREST STATEMENT

The authors conducted the present study with a research grant from PST Inc. The hydrogen and air bubble jelly administered to the mice was provided by the Shinryo Co. Hiroyuki Toda is an Editorial Board member of *Psychiatry and Clinical Neurosciences Reports* and a co‐author of this article. To minimize bias, they were excluded from all editorial decision‐making related to the acceptance of this article for publication. The remaining authors declare no conflict of interest.

## ETHICS APPROVAL STATEMENT

All procedures were performed in accordance with ARRIVE guidelines and the National Institutes of Health Guide for the Care and Use of Laboratory Animals (NIH Publications No. 8023, revised 1978). All animal experiments were approved by the local Animal Investigation Committee of the National Defense Medical College (Approval numbers: 18008 and 22042).

## PATIENT CONSENT STATEMENT

N/A

## CLINICAL TRIAL REGISTRATION

N/A

## Data Availability

The data that support the findings of this study are available on request from the corresponding author, Minori Koga.
